# Supplementary material for: Lolium perenne apoplast metabolomics for identification of novel metabolites produced by the symbiotic fungus Epichloë festucae
Source: New Phytol. 2020 Apr 13;227(2):559–71. doi: 10.1111/nph.16528 (PMC7317419; doi:10.1111/nph.16528)
Supplement: Supplementary file 1 — Fig. S1 Principal component analysis (PCA) of 203 metabolite features. Fig. S2 Extracted ion chromatogram of peramine. Fig. S3 Extracted ion chromatogram of epichloëcyclins A–E. Fig. S4 Confirmation of the identity of epichloëcyclins A–E. Fig. S5 gigA gene cluster organization and sequences. Fig. S6 Extracted ion chromatogram of a putative peptide. Fig. S7 LgsA predicted domain structure and multiple sequence alignment. Fig. S8 lgsA deletion and complementation construct design and strain screening. Fig. S9 Culture morphology of WT and ∆lgsA strains. Fig. S10 Host interaction and cellular phenotypes of L. perenne infected with WT and ∆lgsA strains. Fig. S11 ESI‐HR‐MS/MS fragmentation of 472 m/z metabolite in negative mode. Fig. S12 472 and 308 metabolite isotope analysis. Fig. S13 Strategy for deletion of E. festucae lgsC, Southern blot and PCR analysis. Fig. S14 Host interaction phenotype of L. perenne infected with WT and ∆lgsC strains. [file NPH-227-559-s001.pdf]

New Phytologist

***New Phytologist* Supporting Information**

Article title: *Lolium perenne* apoplast metabolomics for identification of novel metabolites produced by the symbiotic fungus *Epichloë festucae*

Authors: Kimberly A. Green, Daniel Berry, Kirstin Feussner, Carla J. Eaton, Arvina Ram, Carl H. Mesarich, Peter Solomon, Ivo Feussner and Barry Scott

Article acceptance date: 28 February 2020

The following Supporting Information is available for this article:

a

b

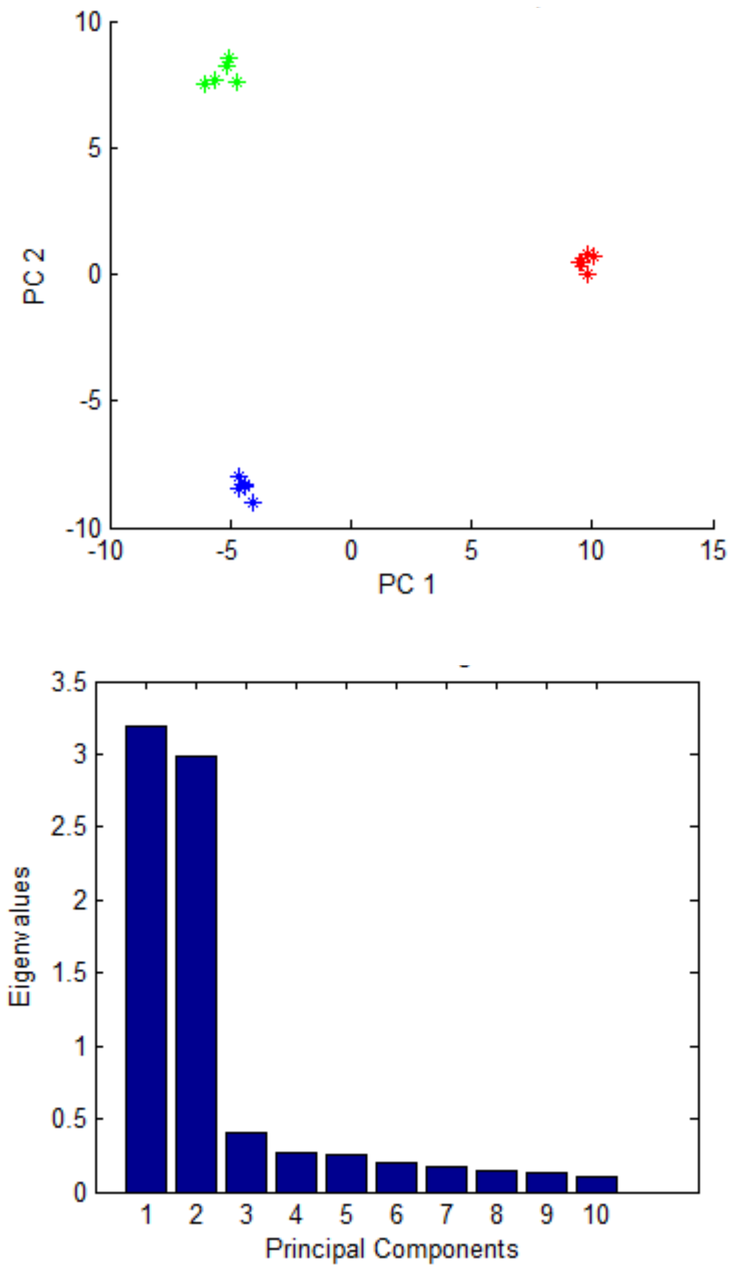

Fig. S1 Principle component analysis (PCA) of 203 metabolite features with a false discovery rate of < 0.003, obtained by metabolite fingerprinting using ultra-performance liquid chromatography-electrospray-ionisation-time-of-flight mass spectrometry (UPLC-ESI-TOF-MS) analysis of apoplastic wash fluids from mock-treated (blue), or FI1 (red)- or CT (green)-infected *L. perenne*. (a) PCA scores plot and (b) PCA eigen values are shown. Five biological replicates per condition were used for the analysis.

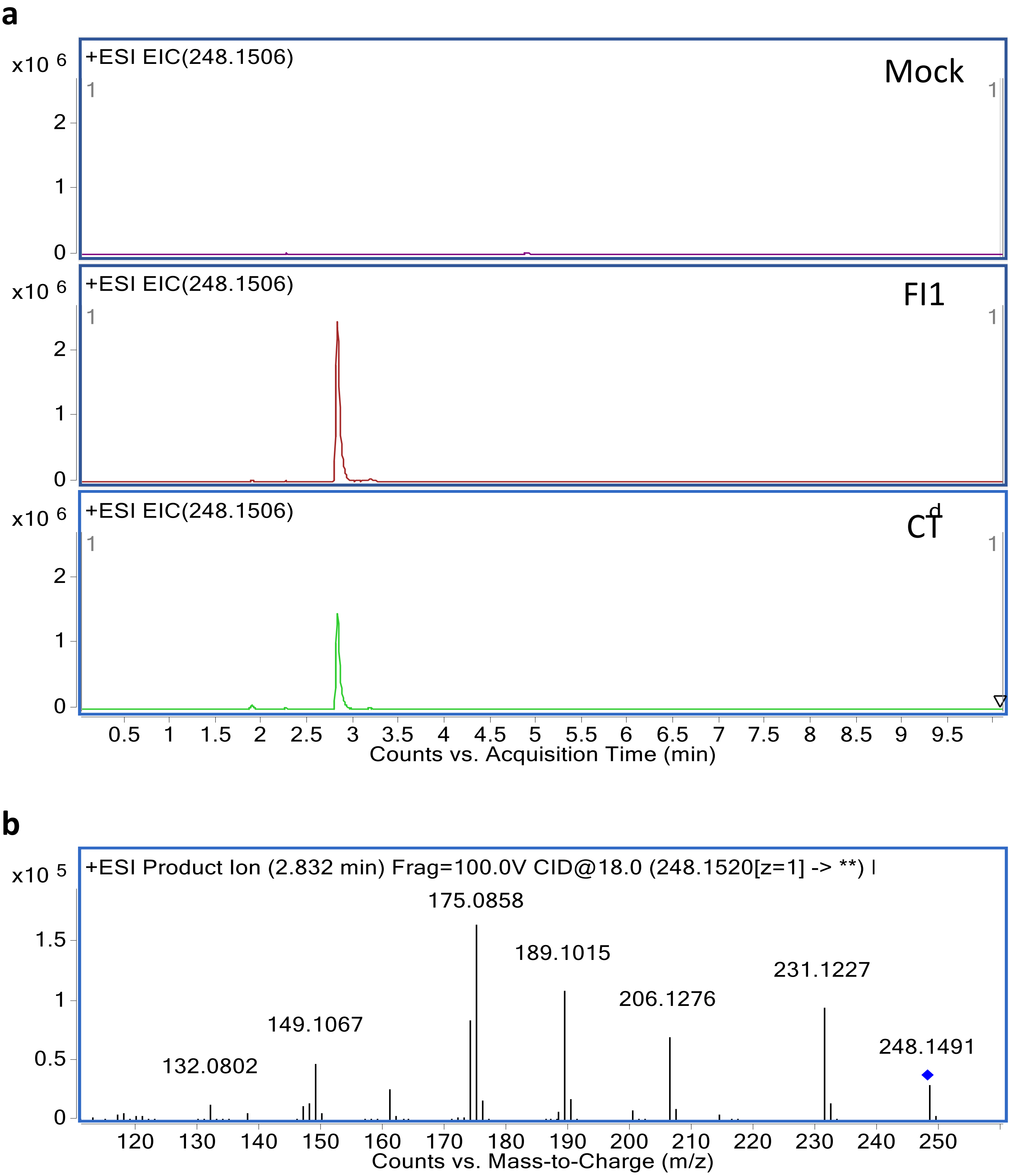

Fig. S2 Extracted ion chromatogram of peramine ($[M+H]^+$ 248.1506) in Fl1 and CT (a) and confirmation of the peramine structure by ultra high-performance liquid chromatography-

quadrupole time-of-flight mass spectrometry/mass spectrometry (UHPLC-QTOF-MS/MS) analysis. (b) Data are representative for five biological replicates per condition.

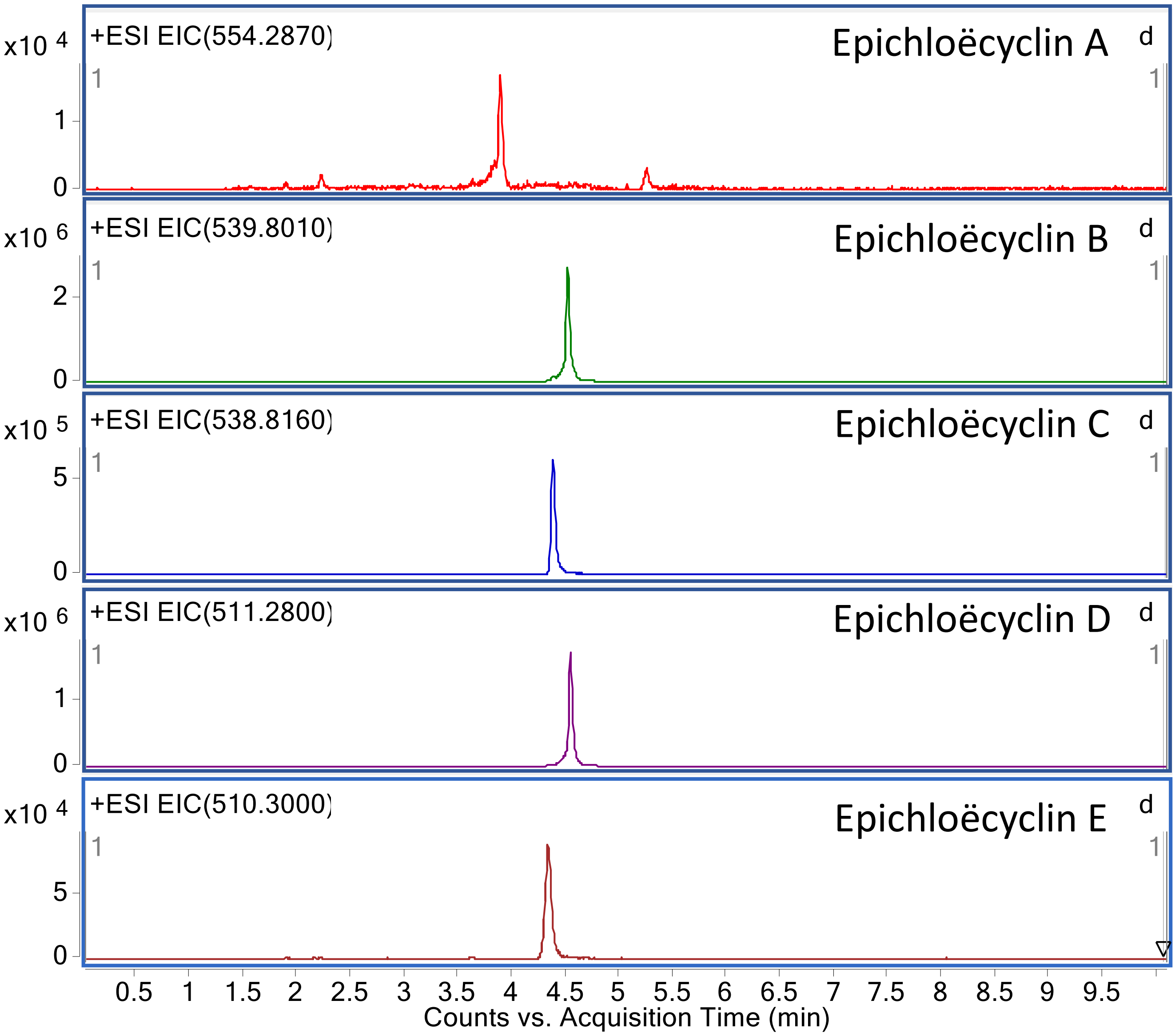

Fig. S3 Extracted ion chromatogram of epichloëcyclins A-E, obtained by ultra high-performance liquid chromatography-quadrupole time-of-flight-mass spectrometry (UHPLC-QTOF-MS) analysis of Fl1 samples. Data are representative for five biological replicates per condition.

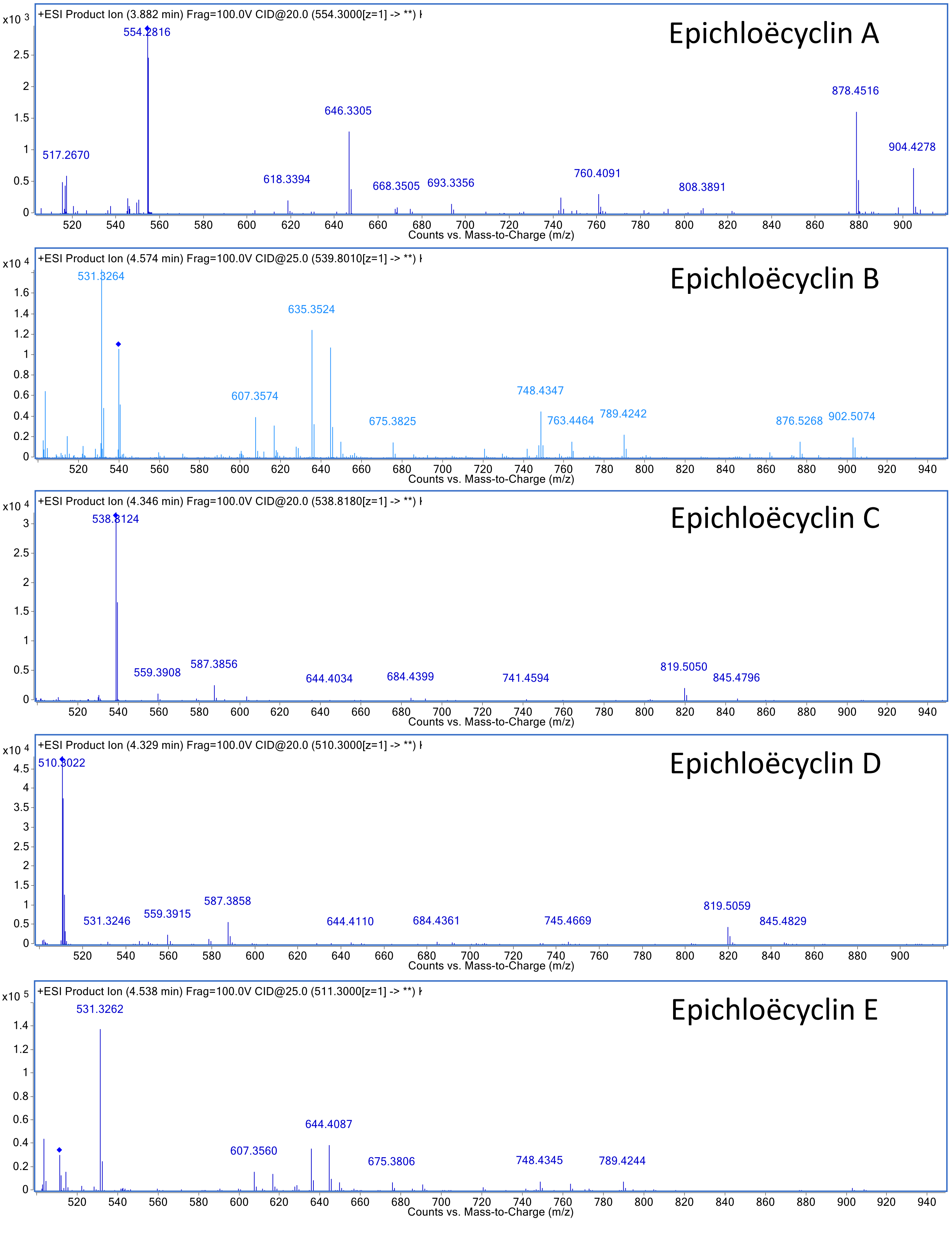

Fig. S4 Confirmation of the identity of epichloëcyclins A-E by ultra high-performance liquid chromatography-quadrupole time-of-flight-mass spectrometry/mass spectrometry (UHPLC-

QTOF-MS/MS) analysis of the ions $[2M+H]^{2+}$. The fragment spectra shown here for epichloëcyclins A-E match the spectra described by **Johnson RD, Lane GA, Koulman A, Cao M, Fraser K, Fleetwood DJ, Voisey CR, Dyer JM, Pratt J, Christensen M, et al. 2015.** A novel family of cyclic oligopeptides derived from ribosomal peptide synthesis of an *in planta*-induced gene, *gigA*, in *Epichloë* endophytes of grasses. *Fungal Genetics and Biology* **85**: 14-24.

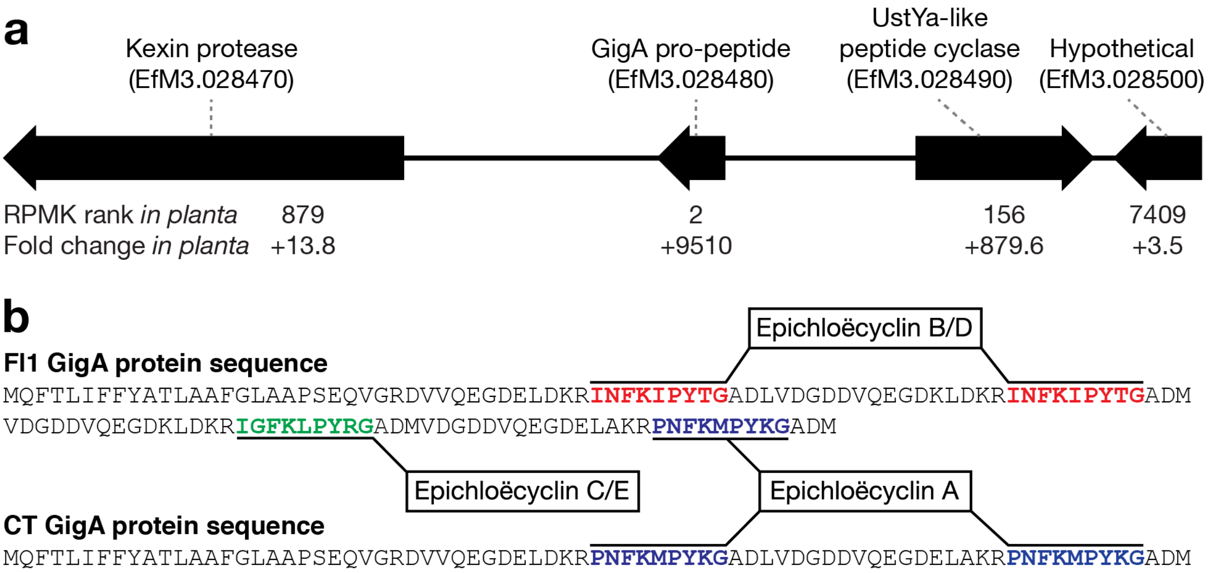

Fig S5 *gigA* gene cluster and GigA protein sequences. (a) The *gigA* gene cluster annotated with gene expression level (ranked highest to lowest for all *E. festucae* genes expressed *in planta*) and fold change in expression *in planta* compared to axenic culture. The transcriptome data used here is available from the Sequence Read Archive (SRA) under Bioproject PRJNA447872. (b) GigA pro-peptide sequences from *E. festucae* strains Fl1 and CT annotated with the epichloëcyclin repeat sequences.

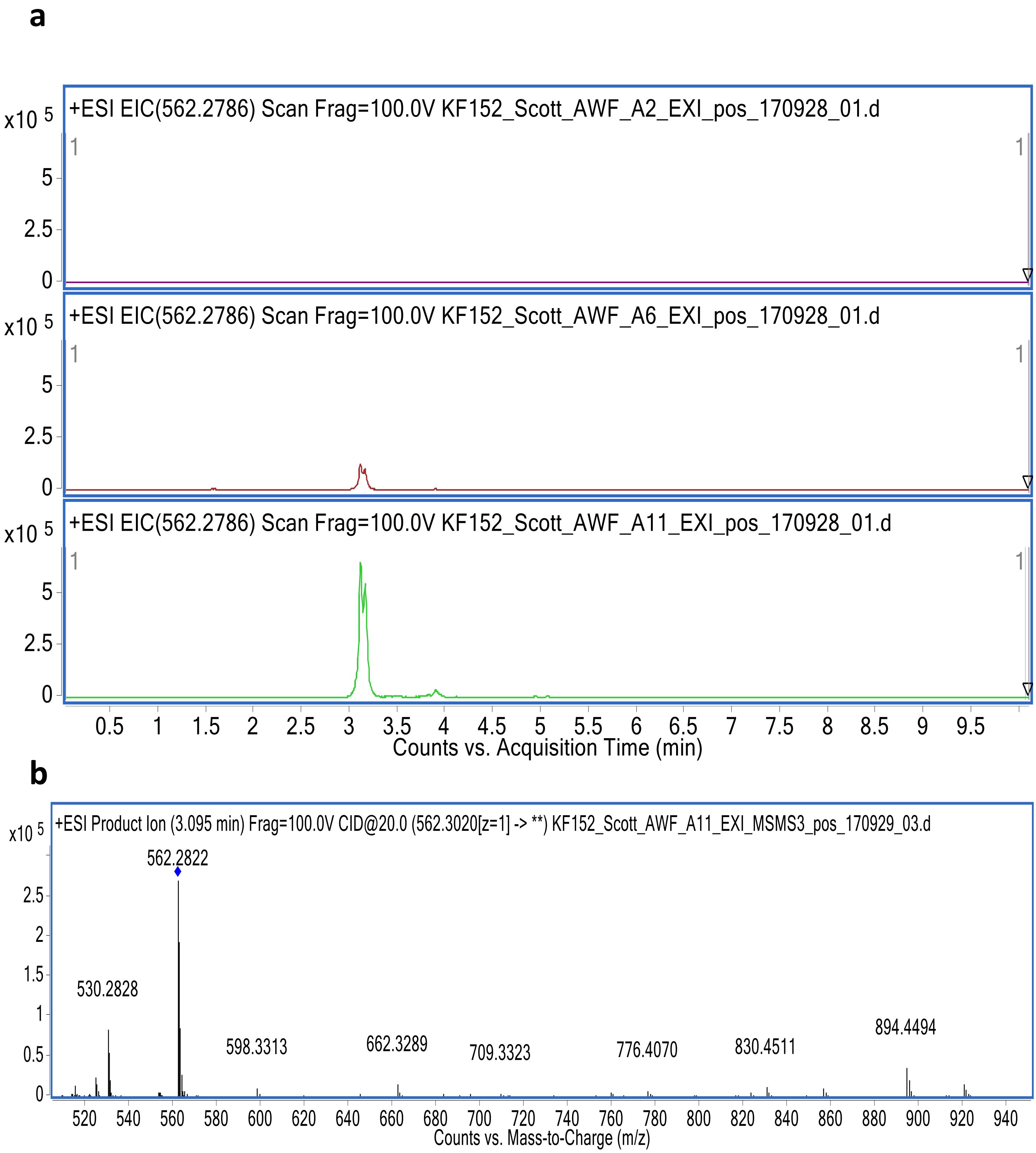

Fig. S6 Extracted ion chromatogram of a putative peptide of $[2M+H]^{2+}$ 562.2786 (put. peptide 1), which is strongly enriched in CT. (a) Data are representative for five biological replicates per condition. (b) The fragment spectrum of this putative peptide 1 obtained by

ultra high-performance liquid chromatography-quadrupole time-of-flight-mass spectrometry/mass spectrometry (UHPLC-QTOF-MS/MS) analysis is shown.

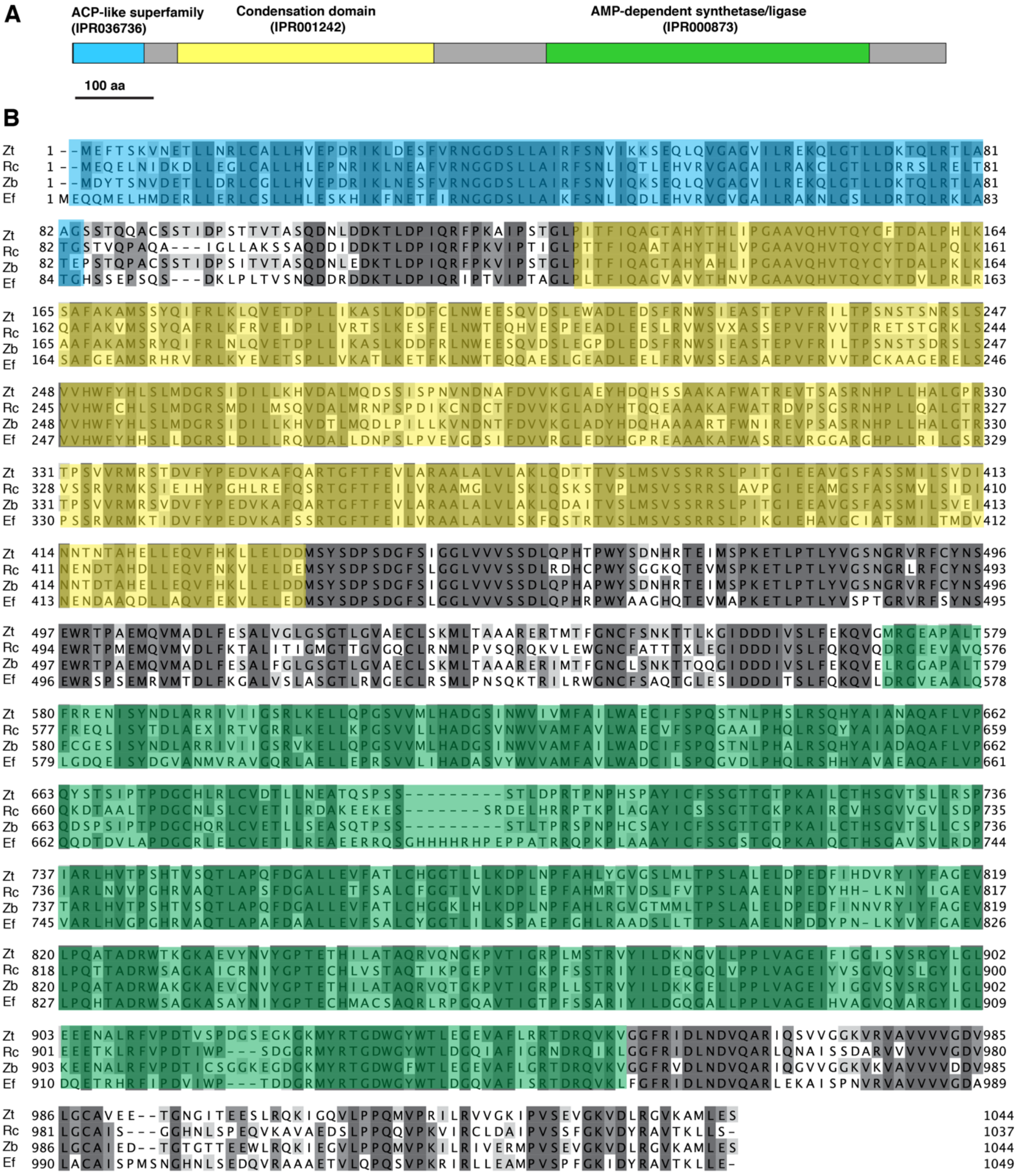

Figure S7 LgsA predicted protein structure and multiple sequence alignment. (a) *Epichloë festucae* LgsA protein structure with respective InterproScan domains as indicated. Bar = 100 amino acids (aa). (b) Multiple sequence alignment of *E. festucae* (Ef) EfM3.056230, *Zymoseptoria tritici* (Zt) ZT1E4_G1886, *Ramularia collo-cygni* (Rc) RCC_03904, *Zymoseptoria brevis* (Zb) TI39_contig403g00012-encoded proteins. Domains as per (a), are indicated.

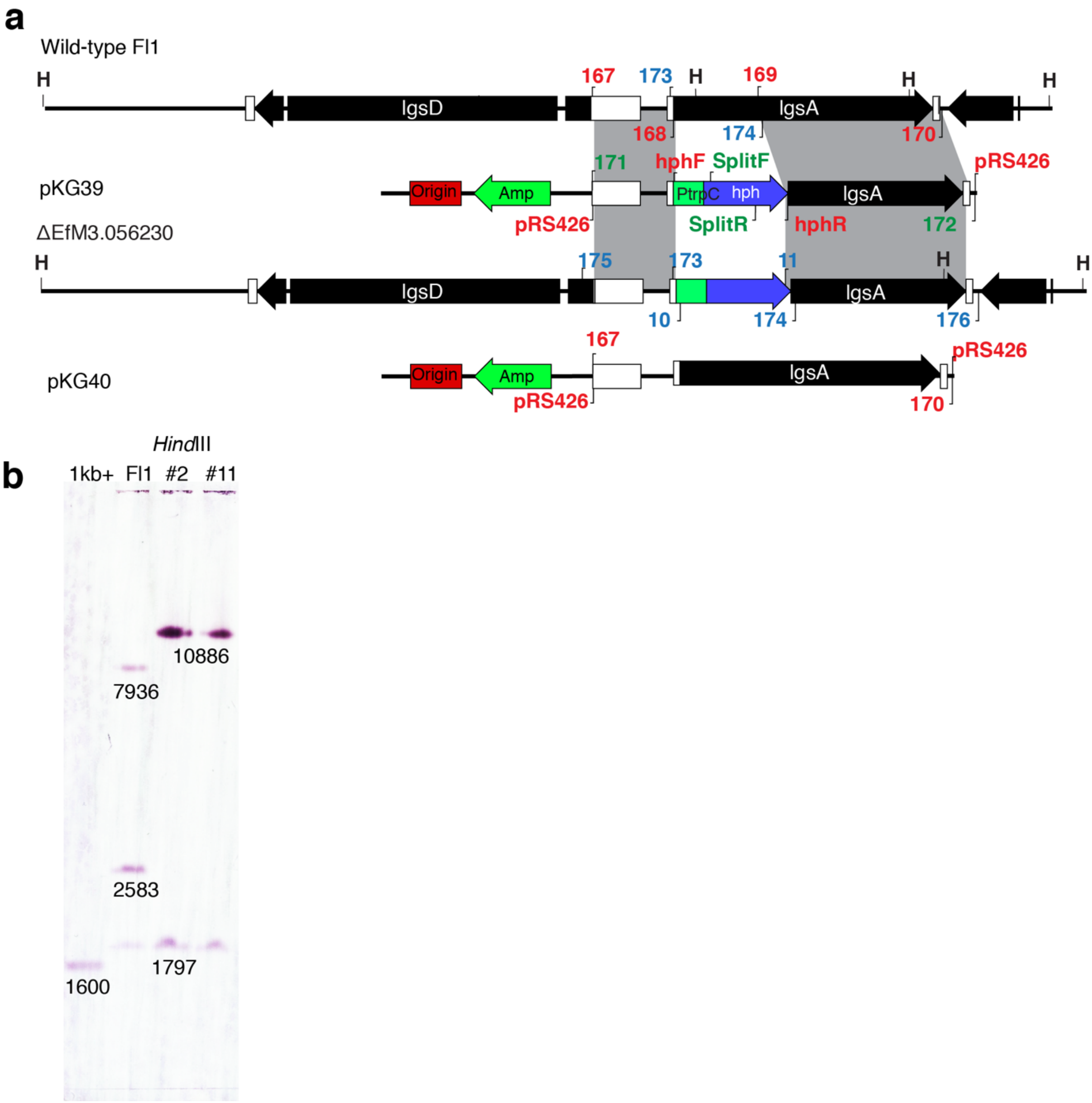

Fig. S8 *lgsA* deletion and complementation construct design and strain screening. (a) Schematic of the WT (Fl1) *lgsA* genomic locus and linear insert of *lgsA* deletion construct pKG39 and complementation construct pKG40. Regions of recombination are indicated by grey shading. *Hin*dIII(H) restriction enzyme sites used for Southern analysis and PCR primers used for Gibson assembly (red), split marker transformations (green) and knock-out screening (blue) are as shown. (b) nitro-blue tetrazolium/5-bromo-4 chloro-3'-indolyphosphate (NBT/BCIP)-stained Southern blot of *Hin*dIII genomic DNA digests (1.5 µg) probed with digoxigenin (DIG)-11-dUTP-labeled linear pKG39 purified PCR fragment (primers 171/172). Fragments of the expected size are as shown.

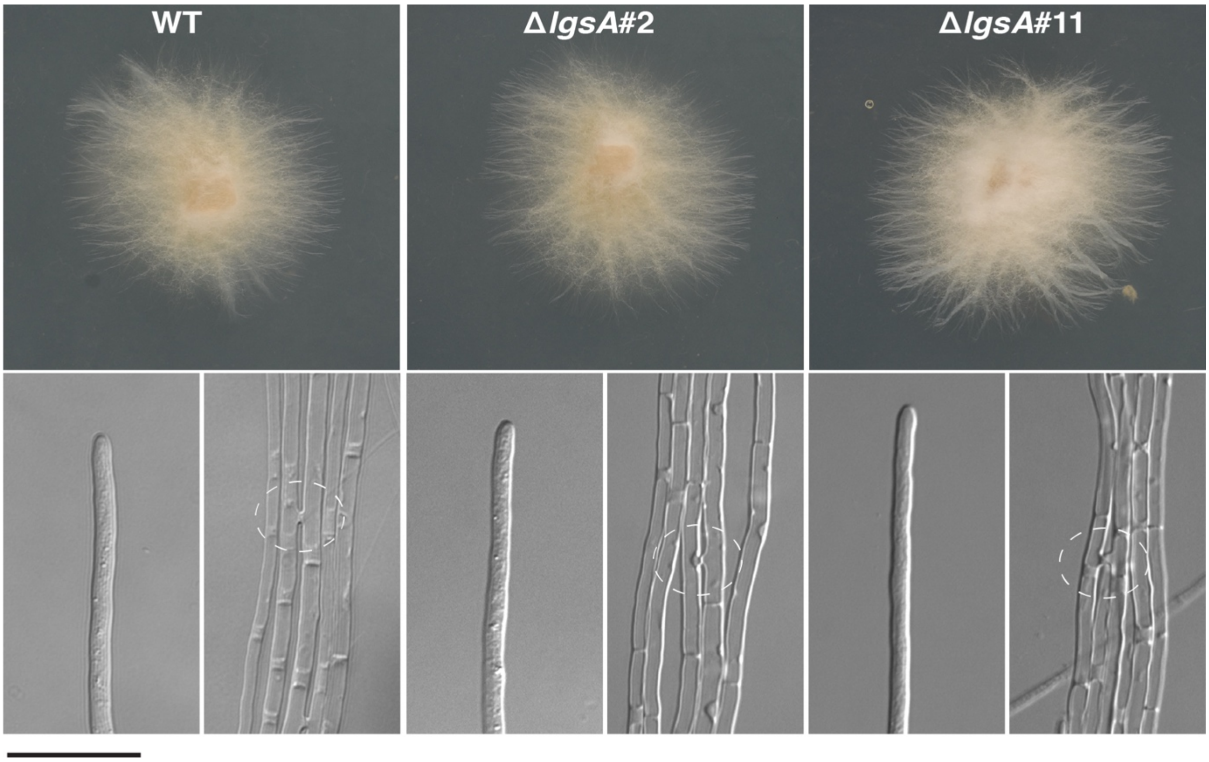

Fig. S9 Culture morphology of WT and Δ*lgsA* strains. Culture morphology of WT and Δ*lgsA* strains grown on PD agar for seven days at 22°C (upper row). DIC images of hyphae undergoing cell-cell fusion on 1.5% water agar (highlighted by broken circles) in lower row. Bar = 20 µm.

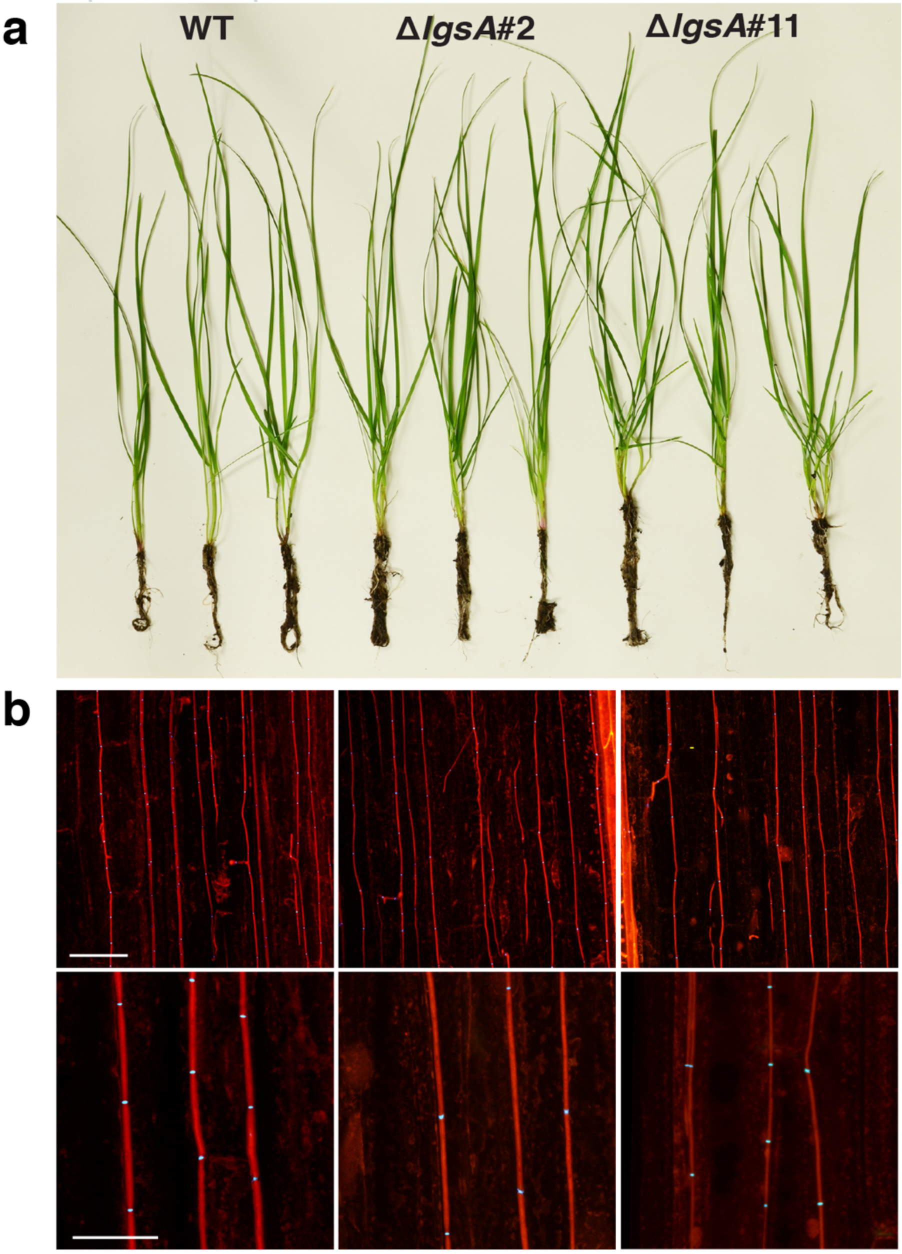

Fig. S10 Host interaction and cellular phenotypes of *L. perenne* infected with WT and Δ*lgsA* strains. (a) Host interaction phenotype of *L. perenne* plants infected with WT and Δ*lgsA* strains at 8 weeks post planting. (b) Confocal depth series images of *L. perenne* leaf sheaths infected with WT and Δ*lgsA* strains. Samples were stained with aniline blue, which detects β-glucans (red) and wheat germ agglutinin-alexa fluor 488 (WGA-AF488) which detects chitin (blue). Bar = 25 µm.

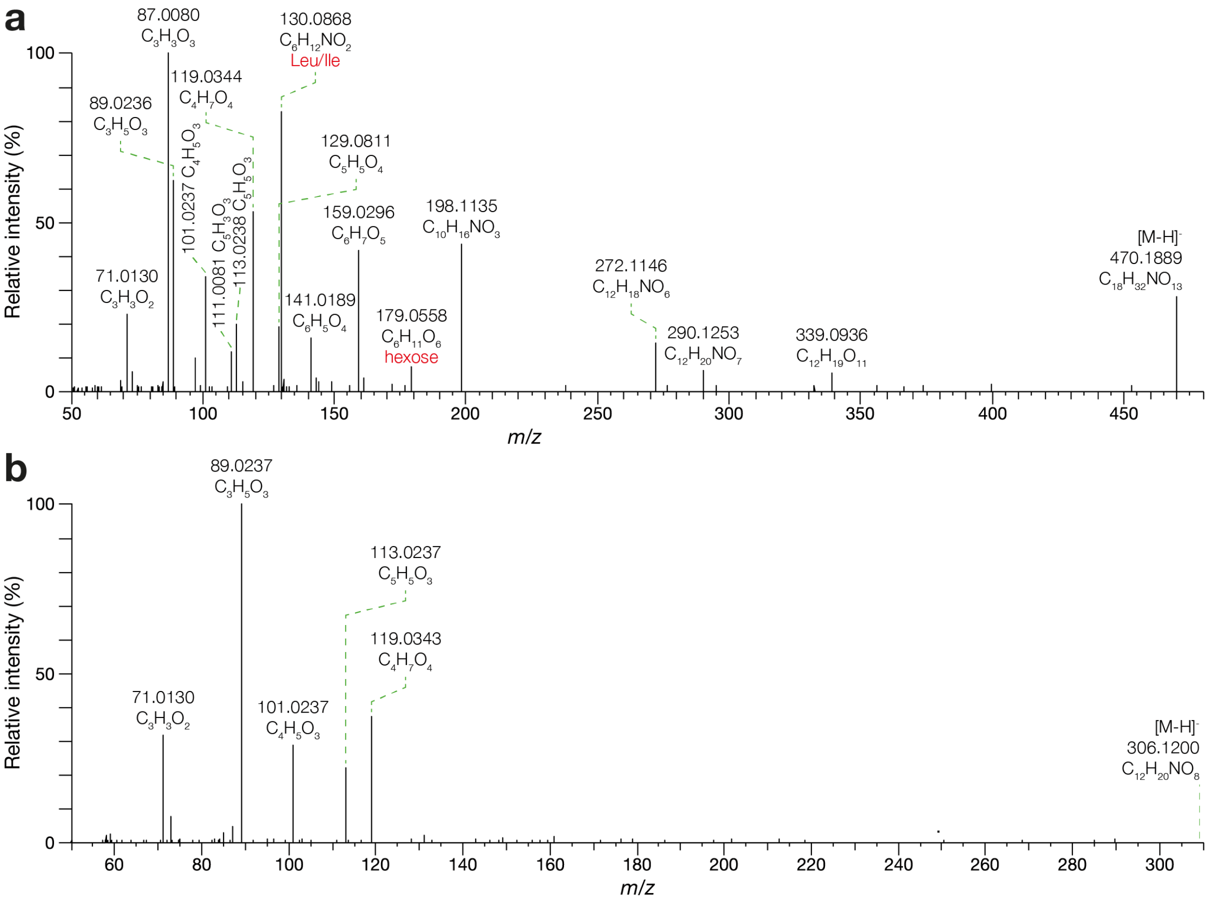

Fig. S11 Negative mode ESI-MS/MS spectra. (a) High-resolution accurate mass negative electro spray ionisation-mass spectrometry/mass spectrometry (ESI-MS/MS) spectrum of the 470 *m/z* *E. festucae* LGS precursor ion fragmented by higher-energy collisional dissociation (HCD) at 20% energy. (b) High-resolution accurate mass negative ESI-MS/MS spectrum of the 306 *m/z* putative *Z. tritici* LGS precursor ion fragmented by HCD at 20% energy.

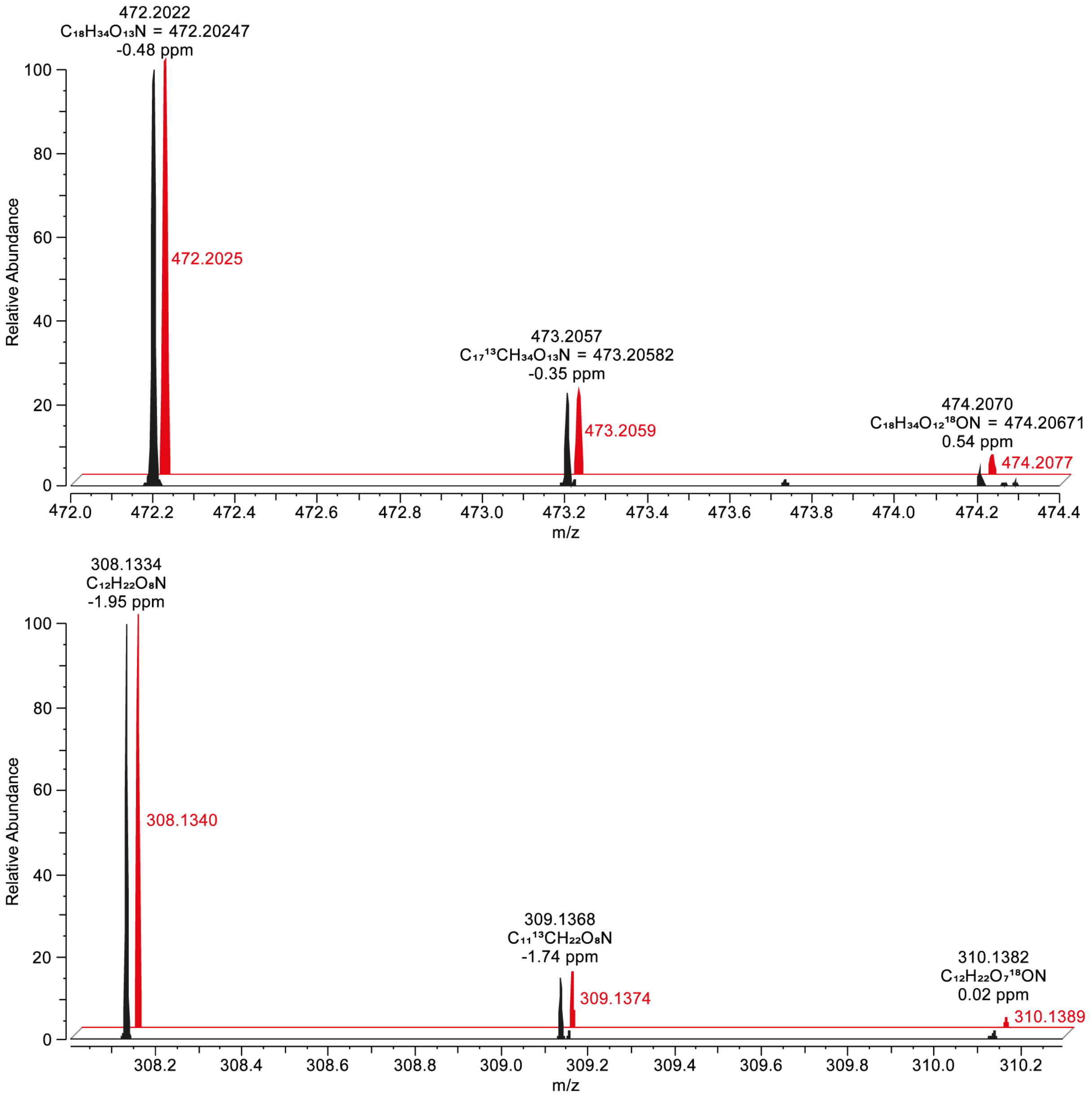

Fig. S12 Observed (black) and simulated (red) isotope distributions for the 472 *m*/*z* (top) and 308 *m*/*z* (bottom) metabolites assuming molecular formulas of $C_{18}H_{33}O_{13}N$ + H^+ and $C_{12}H_{21}O_8N + H^+$, respectively. Isotope distribution simulations were performed using Thermo FreeStyle 1.6.

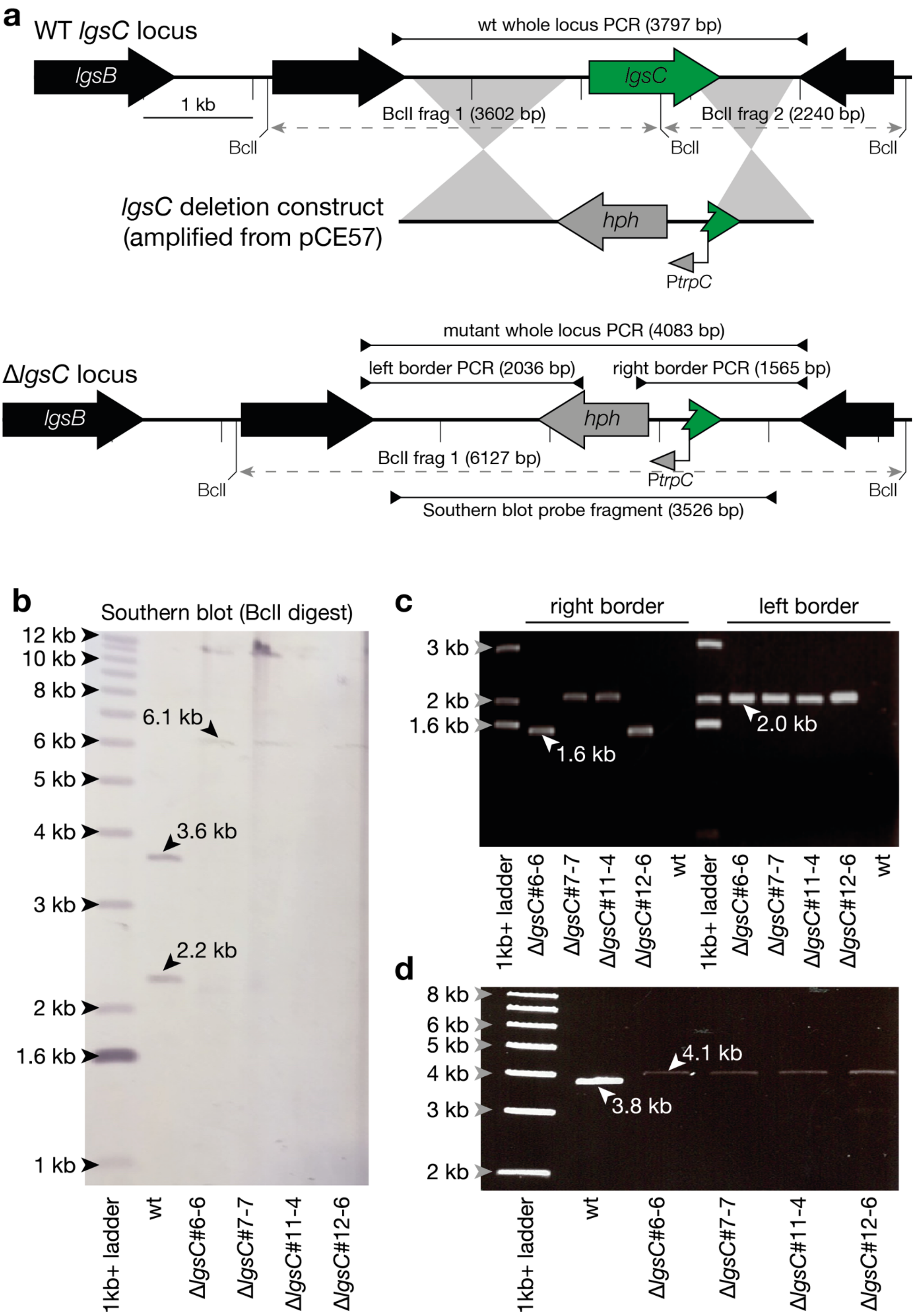
a
WT lgsC locus
wt whole locus PCR (3797 bp)
lgsB
lgsC
1 kb
BclI frag 1 (3602 bp)
BclI frag 2 (2240 bp)
BclI
BclI
BclI
lgsC deletion construct
(amplified from pCE57)
hph
PtrpC
mutant whole locus PCR (4083 bp)
ΔlgsC locus
left border PCR (2036 bp)
right border PCR (1565 bp)
lgsB
hph
BclI frag 1 (6127 bp)
PtrpC
BclI
BclI
Southern blot probe fragment (3526 bp)
b
Southern blot (BclI digest)
12 kb
10 kb
8 kb
6.1 kb
6 kb
5 kb
4 kb
3.6 kb
3 kb
2.2 kb
2 kb
1.6 kb
1 kb
1kb+ ladder
wt
ΔlgsC#6-6
ΔlgsC#7-7
ΔlgsC#11-4
ΔlgsC#12-6
c
right border
left border
3 kb
2 kb
1.6 kb
1.6 kb
2.0 kb
1kb+ ladder
ΔlgsC#6-6
ΔlgsC#7-7
ΔlgsC#11-4
ΔlgsC#12-6
wt
1kb+ ladder
ΔlgsC#6-6
ΔlgsC#7-7
ΔlgsC#11-4
ΔlgsC#12-6
wt
d
8 kb
6 kb
5 kb
4 kb
3 kb
2 kb
4.1 kb
3.8 kb
1kb+ ladder
wt
ΔlgsC#6-6
ΔlgsC#7-7
ΔlgsC#11-4
ΔlgsC#12-6

Fig. S13 Strategy for deletion of *E. festucae lgsC*, Southern and PCR analysis. (a) Schematic of the *E. festucae* Fl1 (wild-type, WT) *lgsC* genomic locus, the linear *lgsC* deletion construct amplified from plasmid pCE57 using primers pRS426-cpsA-F and cpsA-pRS426-R, and the Δ*lgsC* genomic locus. WT and mutant loci are annotated with *Bcl*I cut sites and amplified regions to aid interpretation of the Southern blot and PCR screening results, respectively. Homologous flanking regions enabling targeted recombination are indicated by grey shading. (b) nitro-blue tetrazolium/5-bromo-4 chloro-3'-indolyphosphate (NBT/BCIP)-stained Southern blot of *Bcl*I-digested genomic DNA (1.5 µg per lane) probed with digoxigenin (DIG)-11-dUTP-labeled linear pCE57 purified PCR fragment (primers AR84/AR85). Fragments of the expected size are as shown. (c) Gel photo showing products of PCR amplification across the left (primers cps8/TC45) and right (TC44/cps7) flanking regions used to mediate integration at the target locus in four putative Δ*lgsC* strains. No amplification of the WT control is expected. (d) Gel photo showing products of PCR amplification (primers cps7/cps8) across the entire Δ*lgsC* integration site. Amplification of *lgsC* is expected for the WT control.

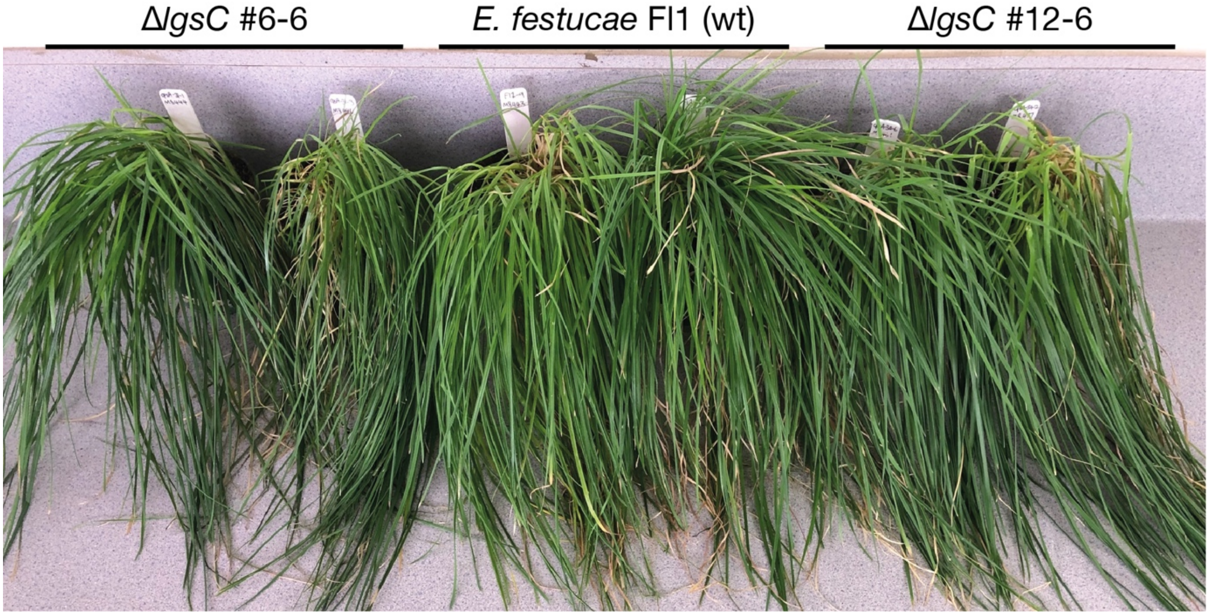

Fig. S14 Host interaction phenotype of *L. perenne* infected with WT and Δ*lgsC* strains. Host interaction phenotype of *L. perenne* plants infected with WT and Δ*lgsC* strains #6-6 and #12-6 several months post-planting.

Table S1 Biological material.

Table S2 Primers used in this study.

Table S3 Data matrix of raw data obtained by UPLC-ESI-TOF-MS-based metabolite fingerprinting analysis of the polar extraction phase, analysed in positive ESI-mode**.**

Table S4 Data matrix of raw data obtained by UPLC-ESI-TOF-MS-based metabolite fingerprinting analysis of the polar extraction phase, analysed in negative ESI-mode.

Table S5 Data matrix of raw data obtained by UPLC-ESI-TOF-MS-based metabolite fingerprinting analysis of the non-polar extraction phase, analysed in positive ESI-mode.

Table S6 Data matrix of raw data obtained by UPLC-ESI-TOF-MS-based metabolite fingerprinting analysis of the non-polar extraction phase, analysed in negative ESI-mode.

Table S7 Data matrix of 203 high quality metabolite features (false discovery rate < 0.003) obtained by metabolite fingerprinting (UPLC-ESI-TOF-MS analysis) of apoplastic wash fluids from mock-treated, Fl1- or CT-infected *L. perenne*.

Table S8 Infection markers identified by metabolite fingerprinting (UPLC-ESI-TOF-MS analysis) and verified by UHPLC-ESI-QTOF-MS/MS analysis or coelution.

Table S9 *Epichloë* metabolite database

New Phytologist

Table S10 Differences in expression of *lgs* cluster genes *in planta* compared to axenic culture.
